# Screening of a lignin decomposing bacterium and its application in bamboo biomechanical pulping

**DOI:** 10.1371/journal.pone.0326076

**Published:** 2025-06-25

**Authors:** Yan Li, Jia Zhang, Juan Li, Ling You, Tao Wang, Zhige Tian, Wenhao Chen

**Affiliations:** 1 Faculty of Agriculture, Forestry and Food Engineering of Yibin University, Yibin, Sichuan, China; 2 Faculty of Economics and Business Administration of Yibin University, Yibin, Sichuan, China; Dr Rammanohar Lohia Avadh University, INDIA

## Abstract

Bamboo is an excellent raw material for papermaking, offering advantages such as a simple papermaking process, abundant availability, short growth cycle, and significant ecological effect. However, the lignin content in bamboo greatly restricts its effective utilization of bamboo pulp. In this study, a strain of SF-6, an efficient ligninolytic bacterium, was screened from bamboo rat feces under restrictive culture conditions and identified as *Enterobacter sichuanensis* by 16S rDNA. The main factors affecting the fermentation of SF-6 was determined by a one-way test, and the optimal culture temperature of the strain was 36.8 °C. The inoculum amount and pH value were determined by response surface analysis. The optimum culture temperature was 36.8 °C, the inoculum amount was 7.5%, and the initial pH was 5.2. Under these conditions, the decomposition rate of lignin was 38.59%, which was 55.42% higher than that before optimization, the paper tear resistance was improved by 73.2%, the breakage strength was improved by 62.41%, and the amount of alkali used was only 1.5 times that of the traditional chemical method. In conclusion, bamboo pulping with SF-6 resulted in a good pulping performance, less energy consumption, and no harm to the environment. Therefore, this is a feasible preparation method. This study provides a new microbial source for lignin degradation.

## Introduction

China is a large consumer of paper products; however, owing to the lack of wood resources, a large number of non-wood materials have been developed and used in the paper industry [1]. Bamboo, as a perennial plant of the bamboo subfamily of the Gramineae family, is unique in the plant field because of its remarkable growth momentum and speed, and is known as the “growth champion” in the plant field [2]. Given the increasingly tight supply of wood pulp and increasing imports, the feasibility of using bamboo pulp as a potential substitute has become increasingly prominent [3]. The main components of bamboo are cellulose, hemicellulose, and lignin. The structural complexity of lignin is a key factor restricting the effective utilization of bamboo pulp [4]. The physical, chemical, and synthetic equipment required for the traditional lignin pretreatment process are expensive and can easily produce pollution [5]. Therefore, effectively overcoming these problems and improving the utilization rate of bamboo pulp are essential research directions.

The biodegradation of lignin has attracted a great deal of attention in the past because of its low energy consumption and environmental friendliness [6]. Microorganisms produce a variety of lignin-degrading enzyme systems, degrade lignin through a synergistic action between enzyme systems, and degrade macromolecular lignin polymers into small molecular compounds or monomer compounds, with the main products being CO_2_ and H_2_O [7]. Most microorganisms with lignin-degrading functions produce laccase and manganese peroxidase, whereas very few microorganisms produce lignin peroxidase [8]. Studies have shown that fungi, especially white rot fungi, have the strongest lignin degradation abilities compared to bacteria, Actinomyces, and fungi [9]. White rot fungi completely decompose lignin into CO_2_ and H_2_O, and are considered the most efficient lignin-degrading microorganisms [10]. However, lignin degradation by fungi is associated with problems such as long pretreatment times, poor environmental adaptability, and spore pollution.

Compared with fungi, lignin-degrading bacteria have become promising candidates due to their shorter culture period, genetic adaptability, phenotypic plasticity and ability to degrade a wider range of intractable pollutants [9]. Lignin-decomposing bacteria produce fewer inhibitors and have selective effects, preventing the partial loss of cellulose. Therefore, the use of lignin-decomposing bacteria may increase the possibility of finding the most cost-effective way to treat lignin. Although some bacteria with lignin-decomposing characteristics have been described, due to their inefficient lignin-increasing ability, better lignin-decomposing bacteria still need to be explored. In addition, it is reported that many bacterial species and their enzymes have the functions of lignin degradation, decolorization and toxicity reduction, but the reduction rate of contamination parameters by bacterial strains is lower than that of fungi [10]. Therefore, it is necessary to search for potential bacterial strains in order to effectively treat pulp and papermaking wastewater.

Bacteria are much more tolerant to pH, temperature, oxygen, and other environments and are easy to control [11]. It has been reported that bacteria decompose lignin not in the way of mineralization, but through secretory enzymes, by β-aryl ether, 2-phenyl, and other chemical bonds to degrade [12]. However, there are few reports on the degradation of bamboo lignin by bacteria during biopulping. In this study, we have screened and isolated bacteria that efficiently decompose bamboo and wood quality from bamboo and rat feces, determined their classification status by molecular biology and morphological methods, used metabolomic analysis to understand their metabolic processes preliminatively, optimized the culture conditions of the strains by combining single factor and response surface tests to improve their lignin degradation rate, and then applied them to pulp and paper making. This lays the foundation for industrial production.

## Materials and methods

### Materials

Trace element mixture (g/L): FeCl_3_·6H_2_O 0.16, ZnSO_4_·7H_2_O 1.5, CoCl_2_·6H_2_O 0.16, CuSO_4_·5H_2_O 0.15, MnSO_4_·H_2_O 1.5, H_3_BO_3_ 0.3, Na_2_MoO_4_·2H_2_O 0.1; Guaiacol medium (g/L): 1 mL/L, KH_2_PO_4_ 1.0, MgSO_4_·7H_2_O 1.0, peptone 1.0, trace element 1 mL/L; Solid medium (g/L): guaiacol 1 mL/L, KH_2_PO_4_ 1.0, MgSO_4_·7H_2_O 1.0, peptone 1.0, AGAR 15.0, trace element 1 mL/L; Aniline blue medium (g/L): aniline blue 0.1, H_2_PO_4_ 1.0, MgSO_4_·7H_2_O 1.0, peptone 1.0, trace element 1 mL/L, AGAR 15.0; Lignin degradation medium (g/L): alkaline lignin 2.0, KH_2_PO_4_ 1.0, MgSO_4_·7H_2_O 1.0, peptone 1.0, trace element 1 mL/L; Lignin degradation solid medium (g/L): alkaline lignin 2.0, KH_2_PO_4_ 1.0, MgSO_4_·7H_2_O 1.0, peptone 1.0, trace element 1 mL/L, AGAR 15.0; Bamboo stalk fermentation medium: The bamboo stalk part of the two-year bamboo was taken, the segments were removed, the skin was removed, and the bamboo blocks were cut into 3 ~ 5 cm in length, 1 ~ 2 cm in width and 2 ~ 3 mm in thickness. Based on the lignin-degradation medium, 2% (w/v) bamboo blocks were added.

### Isolation and screening of lignin-degrading bacteria

Adult bamboo rats were obtained from the bamboo rat breeding base in Cuiping District, Yibin City, Sichuan Province, China, and fed fresh bamboo for three days. Fresh bamboo rat feces were activated with aseptic distilled water and suspended in shock for 1 h. The obtained supernatant was inoculated into 100 mL conical bottle containing 50 mL guaiacol medium at 10% inoculation rate for static culture at 30 °C for 15 days. After extracting the supernatant with color reaction, the concentration of 1 mL was uniformly coated on the solid medium of guaiacol by diluting the coating plate method, and cultured at 30 °C for 3 days. Colonies with red ring edges were dots on the solid guaiacol and aniline blue media, respectively. After 5 days, the diameters of the colonies and the chromogenic and decolorizing rings were measured, and the average value was obtained three times. Strains with large chromogenic rings and obvious decolorization rings were screened and stored for later use. The procedures for experiments and animal care were approved by Yibin University (Approval Number. 20250103001), and conformed to the Guide for the Care and Use of Laboratory Animals produced by the National Institutes of Health.

### Determination of lignin decomposition rate

An alkaline lignin solution with concentrations of 20, 40, 60, 80, and 100 mg/L was prepared, the OD_280_ value was determined, and a standard absorbance curve was drawn. A linear relationship of Y = 0.1716X-0.0016, R^2^ = 0.9963 was obtained. SF-6 was added into 100 mL lignin degradation medium and cultured at 30 °C and 120 r/min. Samples were collected on days 1, 3, 5, and 7, and centrifugated at 12000 r/min for 10 minutes. The supernatant was filtered through a filter membrane and the filtrate was diluted 30 times with a lignin-free degradation medium (control). The OD values were measured at a wavelength of 280 nm and repeated three times, and the absorption value was input into the regression equation to calculate the lignin degradation rate.

### Electron microscope observation

The microscopic morphology of SF-6 was observed using scanning electron microscopy (SEM). Briefly, the colonies were cultured at 30 °C for 5 days on solid multiscreen medium and placed in 2.5% glutaraldehyde fixing solution at 4 °C overnight. Rinse in 0.2 mol/L phosphate buffer solution (pH 7.2) buffer solution for 3 times at a rate of 10 min each; 30%, 50%, 70%, 90%, and 100% ethanol were used as gradients, and each gradient was dehydrated twice for 15 min. The treated samples were then transferred into 2 mL sterile tube containing 100% ethanol, repeated three times, and sent to Beijing Scientific Co., Ltd. for SEM analysis.

### Bacterial identification

Molecular identification of the SF-6 isolate was performed by 16S rRNA gene sequencing. With reference to the published study [13], SF-6 genomic DNA was extracted, and PCR amplification was performed using 27F (5’-AGAGTTTGATCCTGGCTCAG-3’) and 1492R (5’-GGTTACCTT GTTACGACTT-3’) as primers. Sequencing was performed by Beijing Yandou Technology Co. Ltd.. The 16S rRNA gene was subjected to BLAST using the NCBI GenBank database (http://www.ncbi.nlm.nih.gov/). Sequences were selected based on maximum similarity and aligned using the ClustalW software. MEGA 7 was used to construct a phylogenetic tree.

### Preparation of bacterial suspension

The SF-6 isolate was inoculated in 250 mL triangular bottle containing 100 mL lignin degradation medium and cultured in a shaking table at 30 °C and 120 r/min for 3 days. The number of colonies was determined using the plate-counting method. A bacterial suspension was prepared at a concentration of 1 × 107 CFU/mL.

### Metabolite assay

The SF-6 isolate solution (100 μL) which was filtered by 0.45 μm membrane filter were individually grounded with liquid nitrogen and the homogenate was resuspended with prechilled 80% methanol and 0.1% formic acid by well vortex.The samples were incubated on ice for 5 min and then were centrifuged at 15,000 rpm, 4 °C for 5 min. Some of the supernatant was diluted to a final concentration of 53% in methanol by LC-MS-grade water. The samples were subsequently transferred to a fresh Eppendorf tube and then were centrifuged at 15000 g, 4 °C for 10 min. Finally, the supernatant was injected into the LC-MS/MS system analysis [14].

UHPLC-MS/MS analyses were performed using a Vanquish UHPLC system (Thermo Fisher Scientific, Germany) coupled with an Orbitrap Q ExactiveTM HF mass spectrometer(Thermo Fisher Scientific) at Biozeron Co., Ltd. (Shanghai, China). Samples were injected onto a Hypesil Gold column (100 × 2.1 mm, 1.9μm) using a 17-min linear gradient at a flow rate of 0.2 mL/min. Eluents A (0.1% FA in Water) and B (methanol) were used for the positive polarity mode. Eluents A (5 mM ammonium acetate, pH 9.0) and B(methanol) were used for the negative polarity mode. The solvent gradient was set as follows: 2% B, 1.5 min; 2−100% B, 12.0 min; 100% B, 14.0 min; 100−2% B, 14.1 min; 2% B, 17 min. Q ExactiveTM HF mass spectrometer was operated in positive/negative polarity mode with spray voltage of 3.2 kV, capillary temperature of 320 °C, sheath gas flow rate of 40 arb, and auxiliary gas flow rate of 10 arb.

The raw data files generated by UHPLC-MS/MS were processed using Compound Discoverer 3.1 (CD3.1, Thermo Fisher) to perform peak alignment, peak picking, and quantitation for each metabolite. The main parameters were set as follows: retention time tolerance, 0.2 minutes; actual mass tolerance, 5 ppm; signal intensity tolerance, 30%; signal/noise ratio, 3; and minimum intensity, 100, 000. Peak intensities were normalized to the total spectral intensity. Normalized data were used to predict molecular formulas based on additive ions, molecular ion peaks, and fragment ions. The peaks were matched to the mzCloud (https://www.mzcloud.org/), mzVault, and MassList databases to obtain accurate and relatively quantitative results. Statistical analyses were performed using the statistical software R version R-3.4.3, Python 2.7.6, and CentOS release 6.6. When the data were not normally distributed, normal transformations were attempted using the area normalization method, and these metabolites were annotated using the KEGG database (https://www.genome.jp/kegg/pathway.html).

### Detection of optimal culture conditions

The suspensions were added to the lignin degradation medium at 3% inoculation rate, the initial pH was adjusted to 7.0, and the temperature was set at 10, 20, 30, 40, and 50 °C. Each treatment was repeated three times, and the culture was incubated at 120 rpm for 7 days in order to determine the lignin degradation rate. The suspensions were added to the lignin degradation medium at a 3% inoculation rate, and the pH values were set to 5, 6, 7, 8, and 9. Each treatment was repeated three times, and the culture was performed at 30 °C and 120 r/min for 7 days to detect the lignin degradation rate. The initial pH was adjusted to 7.0, and the inoculated amounts in the culture medium were 3%, 6%, 9%, 12%, and 15%. Each treatment was repeated 3 times, and the culture was performed at 30 °C and 120 r/min for 7 days to detect the lignin degradation rate.

### Box-Behnken

Based on the main factors affecting lignin degradation, pH (A), temperature (B), and inoculation amount (C), Design-Expert 8.0 was used to design 17 experiments with three factor 3 levels and, where N is the lignin degradation rate was taken as the response value to optimize the culture conditions, screen out the optimal culture conditions, and conduct verification.

### Bamboo stalk fermentation test

SF-6 isolate was cultured in a constant temperature humidor for 7 days before optimization using bamboo stalk fermentation medium (3% inoculation volume, pH 7.0, 30 °C). Referring to Fan’s cellulose determination method [15], a FIWE 3/6 cellulose analyzer was used to determine the degradation rate of bamboo lignocellulose, and the test was repeated three times.

### Determination of pulping indexes and paper parameters of bamboo after fermentation

The bamboo rod obtained after fermentation was mechanically ground, and bamboo fiber was prepared. One hundred samples were randomly selected from the final prepared bamboo fiber samples. The fiber length and diameter of bamboo fiber samples were determined by using a digital microscope, and the length distribution was analyzed to determine the influence of microbial treatment on the fiber length and diameter. The prepared bamboo fiber was made into paper, and 80 g·m-2 paper was made. The breaking strength (GB/T 1539–2007) and tearing properties (GB/T 455–2002 MOD) were measured, and the paper was prepared as CK using traditional chemical methods.

### Statistical analysis

All of the data are expressed as the mean ± standard deviation (SD), and GraphPad Prism 9.0 (GraphPad Software, USA) was used for statistical analyses. Student’s *t-test* was used for comparisons between two groups. Differences among multiple groups were analyzed using a one-way analysis of variance. Differences were considered statistically significant at *P* <0.05.

## Results

### Isolation and screening of the lignin degrading bacteria

**Fig 1:**
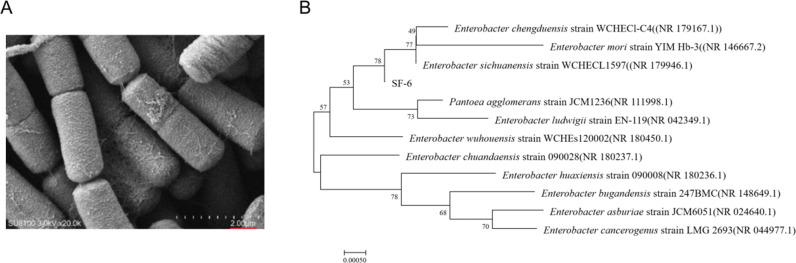
Isolation and screening of the lignin-degrading bacteria. (A) The morphology of IF-6 isolate was observed by scanning electron microscope (SEM). (B) Phylogenetic tree of 16S rRNA sequence of IF-6 isolate.

As shown in Table 1, the SF-1, SF-4, SF-5, and SF-6 isolates obtained from the preliminary screening produced red chromogenic and decolorizing rings. Among them, SF-6 isolate had the most significant effect, and the chromogenic ring on guaiacol solid medium and the fading ring on aniline blue medium were 10.23 mm and 20.40 mm, respectively, indicating that SF-6 isolate had good lassase and peroxidase production activities, and could be used as a follow-up screening strain. In addition, the four isolates were inoculated into the culture medium, and the results showed that they had a good degradation effect on lignin. Among them, SF-6 showed the most obvious effect, with a degradation rate of 23.59% on the 5^th^ day, indicating that this strain had the strongest lignin degradation ability among the tested strains and could be used as a follow-up experimental strain (Table 2). SEM results showed that the SF-6 isolate grew rapidly and was gram-negative, spore-free, capsule-free, and flagellated throughout the body. The size of the isolates was (0.4–0.6) × (1.0–3.0) microns, and the morphology of the isolates was blunt, which was typical of *Enterobacterium* (Fig 1A). The 16S rRNA sequence length of the SF-6 isolate was 1540 bp, and its GenBank accession number was OR647499. Comparison with 11 strains published in GenBank and phylogenetic analysis showed that the SF-6 isolate was closest to *Enterobacter sichuanensis* strain WCHECL1597 (Fig 1B). Based on colony observations, Gram staining, 16S rRNA gene sequencing, and physiological and biochemical data, SF-6 isolate was identified as *Enterobacter sichuanensis*.

**Table 1:** Diameters of the Guaiacol Solid Medium Coloring Circles of Screened Strains and Decolorized Circles in Aniline Blue Medium.

Isolates	Color circle diameter (d_1,_ mm)	Colony diameter (d_2_, mm)	Actual diameter of chromosphere (d_1_-d_2_, mm)	Decolorizing ring diameter (d_1,_ mm)	Colony diameter (d_2_, mm)	Actual diameter of decolorizing ring (d_1_-d_2_, mm)
SF-1	22.17 ± 0.67	16.43 ± 0.93	5.73 ± 0.31	30.50 ± 2.10	15.23 ± 0.59	15.27 ± 1.52
SF-2	7.03 ± 0.55	5.23 ± 0.59	1.80 ± 0.17	9.90 ± 1.05	5.20 ± 0.82	4.70 ± 0.36
SF-3	4.23 ± 0.59	4.17 ± 0.47	0.07 ± 0.12	4.03 ± 0.85	3.93 ± 0.60	0.10 ± 0.26
SF-4	30.40 ± 1.93	25.70 ± 1.30	4.70 ± 0.79	47.97 ± 1.45	36.20 ± 0.92	11.77 ± 0.59
SF-5	40.47 ± 2.14	32.40 ± 1.25	8.07 ± 0.90	60.43 ± 3.57	43.17 ± 2.65	17.27 ± 0.93
SF-6	28.30 ± 0.98	18.07 ± 1.40	10.23 ± 0.49	32.73 ± 1.42	12.33 ± 1.72	20.40 ± 0.60
SF-7	24.20 ± 0.82	19.10 ± 1.65	5.10 ± 0.85	34.33 ± 0.76	25.17 ± 1.16	9.17 ± 0.47
SF-8	5.10 ± 0.36	5.00 ± 0.50	0.10 ± 0.17	13.00 ± 0.50	8.10 ± 1.25	4.90 ± 0.75

**Table 2:** Degradation rate of each strain in the lignin degradation medium in different days.

Isolates	Lignin degradation rate (%)			
	1 d	3 d	5 d	7 d
SF-1	2.18 ± 0.16	8.99 ± 0.49	11.63 ± 1.19	11.54 ± 0.62
SF-4	2.81 ± 0.26	9.56 ± 0.52	16.19 ± 0.82	17.23 ± 0.80
SF-5	2.49 ± 0.36	4.40 ± 0.38	7.76 ± 0.44	8.55 ± 0.45
SF-6	3.56 ± 0.25	16.36 ± 0.41	23.59 ± 0.37	24.72 ± 0.45

### Analysis of metabolites of the SF-6 isolate

**Fig 2:**
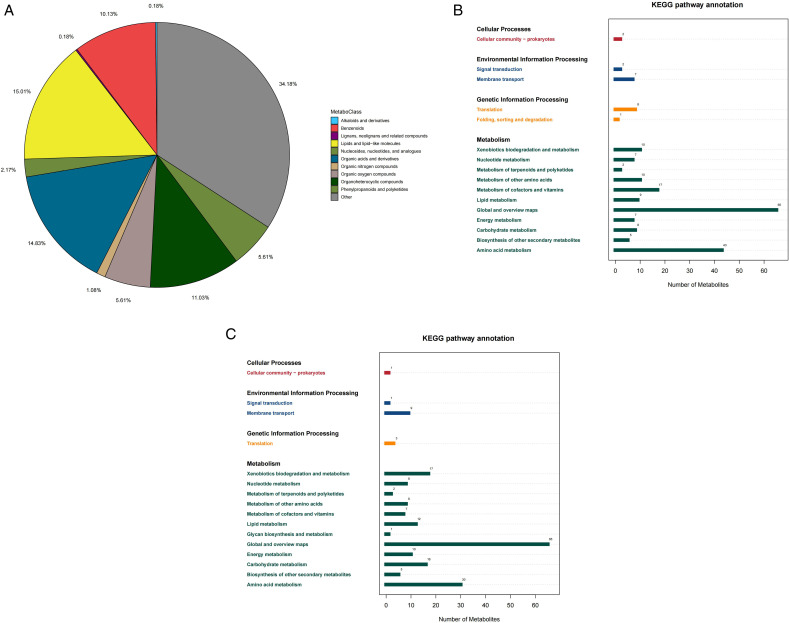
Analysis of the metabolites of the SF-6 isolate. (A) Metabolome sequencing results distribution. (B) Distribution of positive ions in KEGG database. (C) Negative ion distribution in KEGG database.

The metabolome sequencing results are shown in Fig 2A; 553 compounds were detected, including lipids and lip-like molecules (15.01%), organic acids and their derivatives (14.83%), organoheterocyclic compounds (11.03), and benzenoids (10.13%). The positive-ion model detected 353 metabolites, 132 of which were annotated to the KEGG database (Fig 2B). The negative ion model detected 200 metabolites, 114 of which could be annotated to the KEGG database (Fig 2C). The enriched pathways included Cellular Processes, Environmental Information Processing, Genetic Information Processing, and Metabolism.

### Optimal culture conditions for the SF-6 isolate

**Fig 3:**
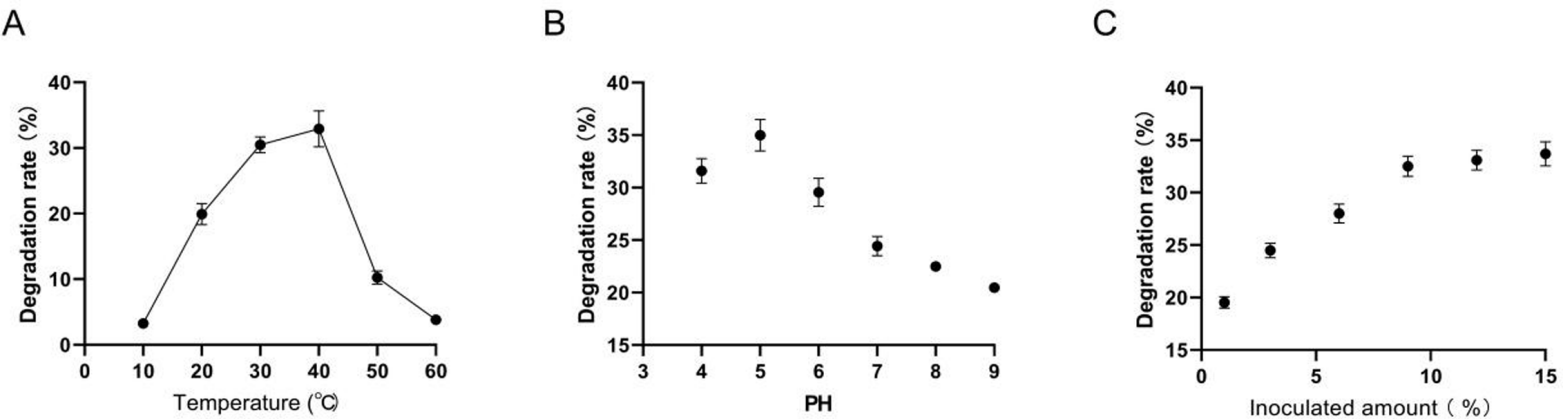
Detection of the optimal culture conditions for SF-6 isolates. (A) Optimum culture time; (B) Optimal culture PH; (C) Optimal inoculated amount.

We detected the optimal culture conditions for SF-6 isolates, and the results showed that the degradation rate of lignin was between 30 and 40 °C, especially at 40 °C, the maximum degradation rate was 32.92% (Fig 3A). Therefore, 40 °C was selected as the culture temperature for the next experiment. The optimal pH value detection results showed that the SF-6 isolates could grow well under PH 4.0-9.0 conditions and had a certain degradation effect on lignin. The degradation rate of lignin was the highest in pH 4.0–6.0, reaching 34.99% (Fig 3B). In addition, as shown in Fig 3C, the lignin degradation rate reached 32.50% when the inoculated amount of SF-6 isolate was 9%. The degradation rate did not increase significantly with an increase in the inoculation amount. Therefore, we selected 40°C, PH 4.0 ~ 6.0, and 9% inoculation volume as the culture conditions for SF-6 isolate.

### Response surface optimization of fermentation conditions

Design Expert 13 software was used to process the test data, and a multiple regression model was established; quadratic polynomial regression equations of temperature (A), initial pH (B), and inoculated amount (C) were obtained: Degradation = 36.72228 + 0.543825A + 1.1756375B + 0.6388375C + 1.806175AB-0.747825 AC-0.6321BC-1.380015A²-3.59479B²-4.14929C².

According to the variance analysis of the regression equation of the model, P < 0.001 indicated a significant difference in the model. The determination coefficient R^2^ was 0.9925, and the adjustment coefficient adjusted R² was 0.9828, which was close, indicating that the regression equation and correlation of the model were good. These results show that the Box-Behnken design is acceptable and can be applied to the theoretical prediction of lignin degradation by SF-6. In addition, F test showed that the main and secondary factors affecting the lignin degradation rate were temperature > inoculum amount >pH. Using Design-xpert software, the maximum degradation rate was obtained when the inoculation amount was 7.53%, the temperature was 36.77 °C, and the pH was 5.25. Under these conditions, the maximum predicted lignin degradation rate was 36.97% (Table 3). These results suggest that the optimal culture conditions are feasible.

**Table 3:** Response surface experimental design and results.

	A: Temperature/°C	B: pH/1	C: Inoculum size/%	Degradation rate/%	Source	Sum of squares	Free degree	Meansquare	F value	P value
1	0	0	0	36.53	A	2.37	9	20.15	102.78	<0.0001
2	1	0	1	31.55	B	11.06	1	2.37	12.07	0.0103
3	−1	0	−1	29.34	C	3.26	1	11.06	56.4	0.0001
4	0	0	0	36.53	AB	13.05	1	3.26	16.65	0.0047
5	1	−1	0	29.68	AC	2.24	1	13.05	66.57	<0.0001
6	1	1	0	35.26	BC	1.6	1	2.24	11.41	0.0118
7	−1	−1	0	31.84	A²	8.02	1	1.6	8.15	0.0245
8	0	1	−1	30.44	B²	54.41	1	8.02	40.9	0.0004
9	0	0	0	37.48	C²	72.49	1	54.41	277.56	<0.0001
10	−1	1	0	30.19	Residual	1.37	1	72.49	369.79	<0.0001
11	−1	0	1	32.32	Lack of Fit	0.6505	7	0.196		
12	0	0	0	36.52	Pure Error	0.7217	3	0.2168	1.2	0.4161
13	0	−1	−1	26.44	Cor Total	182.71	4	0.1804		
14	0	1	1	30.24	R^2^	0.9925	16			
15	1	0	−1	31.55	Adjusted R²	0.9828				
16	0	0	0	36.53	Predicted R²	0.9369				
17	0	−1	1	28.77						

### Bamboo fermentation verification

The results showed that the degradation rates of lignin, cellulose, and hemicellulose in the bamboo slices were 39.64%, 13.43%, and 28.41%, respectively, representing increases of 61.12%, 33.45%, and 41.24%, respectively. After the optimization of SF-6, the weight loss of bamboo was 39.63, which was 61.28% higher than that before optimization. These results show that the lignin degradation rate significantly increased with the optimized SF-6 isolate.

### Effect of the SF-6 isolate on bamboo pulp production by biological process

**Fig 4:**
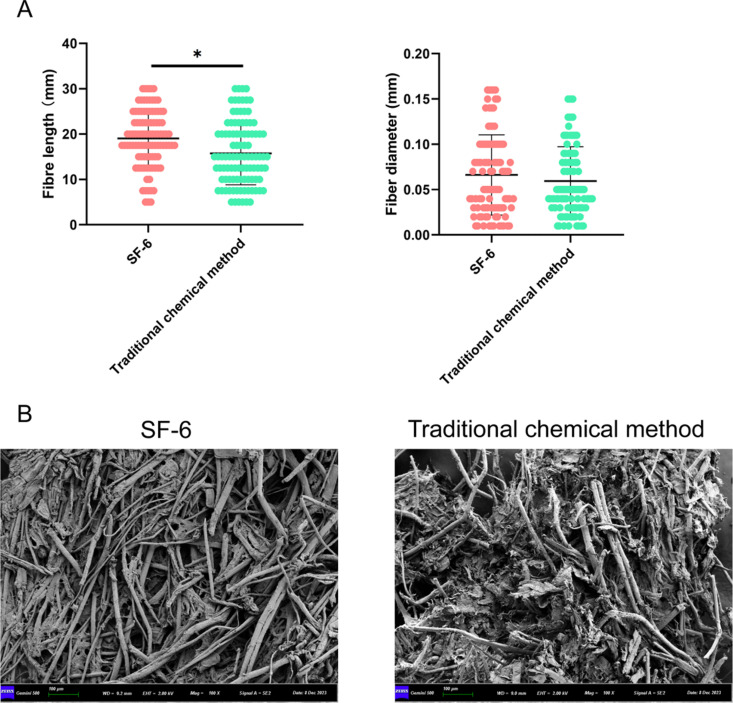
Effect of SF-6 isolate on bamboo pulp production by biological process. (A) Effects of SF-6 isolates on fiber length and diameter. (B) Effects of SF-6 isolates on fiber strength and integrity.

Bamboo was fermented with SF-6 under optimal conditions, and the pulp was prepared. Pulp was prepared using a traditional chemical method as a control group. The results showed that the tearing property and breaking strength of SF-6 treated paper were greatly improved compared with those of the traditional chemical method control group; the tearing resistance was increased by 72.95% and the bursting strength was increased by 63.83%, but the amount of alkali was only 1/6 that of the traditional chemical method (Table 4). To conduct an in-depth analysis of the characteristics of the bamboo pulp fibers, we randomly selected 100 fibers from the prepared fiber samples for testing. The lengths and diameters of the fibers were measured, and the length distribution was statistically analyzed. The results showed that the lengths of the fibers treated with SF-6 ranged from 5 to 54 mm, with an average length of 19.05 mm. The diameters of the fibers treated with SF-6 ranged from 0.01 to 0.16 mm, with an average diameter of 0.0662 mm. However, the average length of the fibers treated with conventional chemical methods was 15.75 mm, and the average fiber diameter was 0.0594 mm (Fig 4A). In addition, the SEM results showed that the fibers treated with SF-6 had higher integrity and fiber strength than those treated with conventional chemical methods (Fig 4B). These results indicate that compared to the traditional chemical pulping process, the bamboo fiber obtained from bamboo fermentation with SF-6 can obtain better properties for making composite materials or high-strength paper.

**Table 4:** Comparison of the paper properties between biological and conventional chemical treatments.

	Alkali charge		Digestion time/h	Digestion temperature/°C	Tearing resistance/mN·m^2^·g^-1^	Bursting strength/ kPa·m^2^·g^-1^	Average fiber length (mm)	Average fiber diameter (mm)
	NaOH/%	Na_2_SO_3_/%						
SF-6	2	1	/	/	26.47 ± 1.08	2.31 ± 0.27	19.05	0.0662
Traditional chemical method	12	6	2	100	15.30 ± 0.55	1.41 ± 0.18	14.75	0.0594

## Discussion

One of the primary components of bamboo is lignocellulose [16]. Because of its high cellulose content and long fiber length, bamboo is one of the preferred raw materials for non-wood papermaking [17]. However, lignin, as one of the components of lignocellulose resources, has been one of the reasons restricting the effective utilization of bamboo lignocellulose due to its complex structure, and it is also the main reason affecting the formation of chroma in the process of papermaking, drying and papermaking wastewater [18,19]. Therefore, the efficient decomposition and removal of lignin from bamboo has become a challenging research topic. Biological pretreatment is widely used to remove lignin from lignocellulosic raw materials because of its low environmental pollution, low equipment requirements and low cost [20,21]. The complete biological decomposition of lignin results from the joint action of bacteria, fungi, and other microorganisms, among which fungi have the most thorough research on lignin decomposition [22]. White rot fungi have been widely studied and used as a model strain for lignin decomposition because of their high efficiency and thoroughness in lignin decomposition [7]. However, owing to their long growth cycles and ease of contamination by miscellaneous bacteria in practical applications, large-scale industrial production is not ideal. In contrast, bacteria can be easily used on a large scale because of their wide range of sources, rapid growth, and strong tolerance to environmental changes [9]. Recently, increasing attention has been paid to the decomposition of lignin by bacteria. Tathagata is derived from the bacterial *Pseudomonas paucimobilis* SYK-6 in soil, which can decompose a variety of basic lignin structures, such as DDVA and syringic acid [23]. *Nasutitermes takasagoensis*, a bacterium isolated from the gut of termites, can decompose 28.0% of non-alkaline lignin [24]. In this study, lignin-decomposing bacteria SF-6 was obtained from the feces of bamboo rats. It was found to have a strong ability to decompose lignin in bamboo, and the degradation rate reached 23.59% on the 5^th^ day of culture.

The culture temperature, pH of the fermentation fluid, and inoculation amount affect the lignin decomposition rate [25,26]. Most of the lignin decomposition pure culture bacteria are medium-temperature microorganisms, suitable for growth temperatures between 30–40 °C [27,28]. The optimum culture temperature after optimization in this study is also 30–40 °C, which is similar to the results of other studies. The key to successful fermentation is the initial pH of the medium and pH control during fermentation [29]. Most lignoenzymes exhibit their optimal state at pH < 7.0 and begin to lose their activity when the pH value is as high as 7.0. In this study, the optimal culture pH of SF-6 isolate was 4.0–6.0, indicating that the lignin decomposition of SF-6 was the best in an acidic PH environment. To investigate the feasibility of SF-6 pulping, we compared the physical properties of pulp and paper using chemical and biological methods. These results showed that the SF-6 isolate could be used in papermaking. The tear performance and breaking strength of SF-6 treated paper were greatly improved compared to those treated by the traditional chemical method. In addition, the SF-6 biological method has higher integrity than the chemical method, and the resulting fiber strength is higher. These findings indicate that SF-6 isolates are effective pulping bacteria with a certain pulping potential. However, its degradation mechanism and differences from other microorganisms and fungi need to be further studied and explored. Moreover, the long pretreatment time of the biopulp process must be reduced.

The present study has limitations. Although we have discovered the effect of SF-6 isolates, there is a lack of sufficient comparison with other microbial ligninolytic systems, which will be conducted in future studies to verify the lignin degradation ability of SF-6 more fully. Furthermore, future studies will employ more characterization techniques to verify the efficacy of SF-6 isolates. Additional, further research on the characteristics, scalability and cost-effectiveness of enzymes in the future is necessary to promote the commercial application of this technology.

## Conclusion

Bamboo pulping with the SF-6 isolate has an acceptable pulping performance, less energy consumption, and causes no harm to the environment. This feasible preparation method provides a new microbial source for lignin degradation. The findings of this study are helpful in reducing the amount of alkali used in the traditional chemical preparation of bamboo pulp. This can not only reduce the cost of wastewater treatment, but also achieve the goal of environmental protection.

## Supporting information

S1 File (RAR)
